# Evaluating Healthcare Claims for Neurocysticercosis by Using All-Payer All-Claims Data, Oregon, 2010–2013

**DOI:** 10.3201/eid2212.160370

**Published:** 2016-12

**Authors:** Robert H. Flecker, Seth E. O’Neal, John M. Townes

**Affiliations:** Oregon Health & Science University, Portland, Oregon, USA

**Keywords:** Neurocysticercosis, cysticercosis, neglected tropical diseases, Taenia solium, Taenia infection, epilepsy, brain disease, parasites, parasitic diseases, helminthiasis, cestode infection, central nervous system diseases

## Abstract

To characterize the frequency of neurocysticercosis, associated diagnostic codes, and place of infection, we searched Oregon’s All Payer All-Claims dataset for 2010–2013. Twice as many cases were found by searching inpatient and outpatient data than by inpatient data alone. Studies relying exclusively on inpatient data underestimate frequency and miss less severe disease.

Neurocysticercosis, central nervous system infection caused by the larval form of the pork tapeworm *Taenia solium*, manifests a broad range of neurologic symptoms, including seizure, headache, obstructive hydrocephalus, encephalitis, stroke, and cognitive disorders (*1*). In the United States, neurocysticercosis is increasingly identified in migrants and travelers from nonindustrialized countries, particularly among the Hispanic population (*2*). However, because no reliable surveillance system for neurocysticercosis exists, studies to document the frequency of the disease have relied primarily on hospital discharge data (*3*–*9*). These studies may underestimate the frequency of neurocysticercosis and provide a biased representation of its effects. In this population-based study of neurocysticercosis, we used the newly implemented All-Payer All-Claims (APAC) database in Oregon, which includes healthcare claims from inpatient, outpatient, and emergency care environments.

## The Study

We analyzed Oregon’s APAC insurance claim data for the 3.5-year period from January 2010 through June 2013. APAC includes claim information collected from most healthcare payers in Oregon and provides a unique person-level identifier allowing analysis at the individual level (*10*). Because no specific code for neurocysticercosis exists, we searched >119 million claim lines for the code for cysticercosis (123.1) from the International Classification of Diseases, Ninth Revision, Clinical Modification (ICD-9-CM), in any of the 13 diagnostic fields at any time during the study period. We extracted all claims from these patients for the entire study period, dropping any claims that were denied to avoid duplication from resubmitted claims. Persons who did not have >1 neurocysticercosis-associated diagnostic, procedure, or current procedural terminology code were also excluded, leaving a list of persons whose condition fit our case definition of neurocysticercosis and all of their paid and capitated claims. We required a supporting diagnostic, procedural, or current procedural terminology code in the case definition to reduce the likelihood of including miscoded cases, while accepting that some true-positive cases may have been excluded. We also randomly selected 4 age- and sex-matched controls from all persons in the dataset, extracted data from all of their claims, and compared the proportion of specific diagnostic codes in neurocysticercosis cases versus codes for controls. We calculated odds ratios (ORs) and 95% CIs by using conditional logistic regression. The denominator for prevalence estimates was taken from US Census Bureau estimates of the insured population in Oregon in 2010.

A total of 137 persons were identified with a 123.1 ICD-9-CM code during the 14 quarters of this study. Of these, 12 were excluded because they did not have paid or capitated claims or did not have an associated neurologic diagnostic or procedural code. The remaining 125 persons whose conditions met the case definition of neurocysticercosis yielded a mean 1-year prevalence of 1.1 cases/100,000 insured persons. Age of case-patients ranged from 3 to 84 (median 41) years. Slightly more cases were found among female patients (69/125, 55%). The 125 cases generated a total of 8,224 claims, of which 2,337 contained a neurocysticercosis-associated neurologic diagnosis or procedure code.

Most claims (5,925/8,224, 72%) were made in outpatient settings. Only 10% (818/8,224) were inpatient claims, and <5% (337/8,224) were generated in emergency departments. Of the 125 persons with neurocysticercosis, 70 (56%) had a claim history that included only outpatient claims, 47 (38%) had both outpatient and inpatient claims, and 3 (2%) had inpatient claims only. Paid and capitated claims associated with mental health disorders, headache, seizure/epilepsy, and syncope were made primarily in outpatient settings, whereas claims associated with hydrocephalus, cerebral edema, and cerebrovascular disease were made primarily in inpatient settings (Figure). Private insurance carriers were the primary payers of paid claims, accounting for ≈55% (4,504/8,224) of payments provided. Federally-funded pay sources provided payment for ≈40% of claims.

**Figure:**
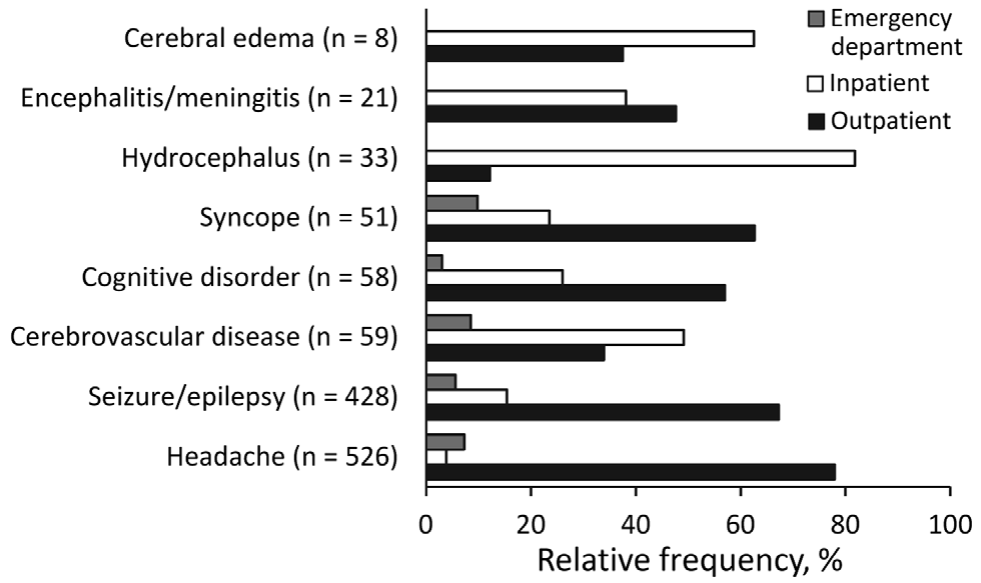
Frequency of neurocysticercosis claims by associated diagnosis and healthcare setting, Oregon, 2010–2013.

Headache was the most common diagnostic category coded in claims for persons with neurocysticercosis (63/125, 50.4%), followed by seizures (41/125, 32.8%); both were significantly more common among case-patients than among controls (OR 3.2, 95% CI 2.1–4.8, and OR 13.3, 95% CI 6.8–25.9, respectively). Other diagnostic codes significantly more common in claims for persons with neurocysticercosis include meningitis/encephalitis, stroke, cognitive disorder, syncope, cerebral edema, and hydrocephalus (Table). The median age for those with a diagnostic code for stroke was significantly lower for those with neurocysticercosis (45 years) than for controls (63 years) (p = 0.05 by Kruskal-Wallis test); no significant differences in age were found for the other diagnostic categories evaluated.

**Table:** Diagnostic codes in healthcare claims from persons with neurocysticercosis (cases) versus age- and sex-matched controls, Oregon, 2010–2013

Diagnostic code group	No. (%) cases, n = 125	No. (%) controls, n = 500	Odds ratio (95% CI)
Meningitis/encephalitis	5 (4.0)	1 (0.2)	20.0 (2.3–171.2)
Seizure	41 (32.8)	19 (3.8)	13.3 (6.8–25.9)
Stroke	17 (13.6)	16 (3.2)	5.6 (2.5–12.4)
Headache	63 (50.4)	120 (24.0)	3.2 (2.1–4.8)
Cognitive disorder	11 (8.8)	16 (3.2)	3.1 (1.4–7.1)
Syncope	12 (9.6)	25 (5.0	2.0 (1.0–4.2)
Psychotic disorder	8 (6.4)	21 (4.2)	1.6 (0.7–3.8)
Anxiety disorder	35 (28.0)	124 (24.8)	1.2 (0.8–1.8)
Mood disorder	32 (25.6)	134 (26.8)	0.9 (0.6–1.5)
Hydrocephalus	7 (5.6)	0	*
Cerebral edema	5 (4.0)	0	*

*Hydrocephalus and cerebral edema were significantly more common among cases. However, the odds ratio could not be calculated because these diagnostic codes did not occur in the control group.

## Conclusions

Assessing the true prevalence or incidence of neurocysticercosis in the United States is difficult due to the lack of active case reporting from providers to public health entities. Most epidemiologic studies, therefore, use hospital-discharge datasets, which capture inpatient cases only (*3*–*9*). The results of our study show that twice as many neurocysticercosis cases are found when inpatient and outpatient data are searched than when only inpatient data are searched. Epidemiologic studies that rely solely on inpatient data largely underestimate the number of cases and the frequency and cost of healthcare interactions for neurocysticercosis.

Although our study detected additional cases of neurocysticercosis that were seen exclusively in the outpatient setting, it still falls far short of capturing all neurocysticercosis cases in Oregon because APAC data do not include the uninsured population. Even though only 14.5% of persons in Oregon lack health insurance (*11*), a previous study involving chart review demonstrated that 40% of neurocysticercosis patients in Oregon were uninsured (*5*). This finding suggests that our study may have missed as many as 80 additional neurocysticercosis cases among the uninsured during the study period.

The predominance of outpatient healthcare claims reflects the growing recognition that neurocysticercosis can result in chronic illness and disability even after the infection has been resolved (*12*–*14*). Patients with neurocysticercosis often require long-term management for disease sequelae such as seizures, hydrocephalus, cerebral edema, and meningoencephalitis, resulting in frequent interaction with specialist providers. This aspect of neurocysticercosis care is not captured in studies based on inpatient data, which tend to highlight acute illness. 

We found that cognitive disorders were coded for 1 of 9 neurocysticercosis patients in this study and were 3 times more likely to occur in these patients than in controls; this finding supports results of recent studies showing that cognitive impairment can be a notable sequela of neurocysticercosis (*12*). Cognitive disorders primarily affect learning, memory, perception, and problem solving. A team-based approach to clinical management that includes ancillary support, including social services and rehabilitation, may be beneficial in neurocysticercosis cases, especially when long-term adherence to antiepileptic drugs or other therapies are required to maintain quality of life. 

Another intriguing finding was that claims coding for stroke were >5 times more likely to appear for patients with neurocysticercosis than for controls and occurred among patients who were significantly younger than controls (data not shown). Although cases of stroke in neurocysticercosis patients have been reported, the effects at the population level have received little attention.

This study has limitations. Although APAC data provided an improved understanding of the neurocysticercosis outpatient population, the data source and quality may introduce bias and limit generalizability. A major limitation of APAC and other healthcare claims datasets is that diagnostic codes associated with a claim may not accurately represent the current clinical context. APAC data are de-identified, which precludes verification of clinical presentation through chart review, a limitation shared by approaches based on other administrative datasets as well. By excluding the uninsured population, this study also underestimates the true prevalence of neurocysticercosis and may present a biased view of diagnostic codes if uninsured cases differ substantially from insured cases. Finally, the composition of neurocysticercosis cases and associated claims in Oregon may not be the same as in other regions of the country.
